# Matrisome Transcriptome Dynamics during Tissue Aging

**DOI:** 10.3390/life14050593

**Published:** 2024-05-07

**Authors:** Zulfiya G. Guvatova, Anastasiya A. Kobelyatskaya, Eveline R. Kudasheva, Elena A. Pudova, Elizaveta V. Bulavkina, Alexey V. Churov, Olga N. Tkacheva, Alexey A. Moskalev

**Affiliations:** 1 Engelhardt Institute of Molecular Biology, Russian Academy of Sciences, 119991 Moscow, Russia; 2 Russian Clinical Research Center for Gerontology, Pirogov Russian National Research Medical University, Ministry of Healthcare of the Russian Federation, 129226 Moscow, Russia

**Keywords:** aging, extracellular matrix, matrisome, RNA-Seq, tissue aging, transcriptome, gene expression

## Abstract

The extracellular matrix (ECM) is a complex three-dimensional network of macromolecules that provides structural support for the cells and plays a significant role in tissue homeostasis and repair. Growing evidence indicates that dysregulation of ECM remodeling contributes to various pathological conditions in the body, including age-associated diseases. In this work, gene expression data of normal human tissues obtained from the Genotype-Tissue Expression project, as well as data from MatrisomeDB 2.0, the ECM-protein knowledge database, are used to estimate the age-dependent matrisome transcriptome dynamics in the blood, heart, brain, liver, kidneys, lungs, and muscle. Differential gene expression (DE) analysis revealed dozens of matrisome genes encoding both structural elements of the ECM and ECM-associated proteins, which had a tissue-specific expression profile with age. Among common DE genes that changed expression with age in at least three tissues, *COL18A1*, *MFAP1*, *IGFBP7*, *AEBP1*, *LTBP2*, *LTBP4*, *LG14*, *EFEMP1*, *PRELP*, *BGN*, *FAM20B*, *CTSC*, *CTSS*, and *CLEC2B* were observed. The findings of the study also reveal that there are sex-specific alterations during aging in the matrisome gene expression. Taken together, the results obtained in this work may help in understanding the role of the ECM in tissue aging and might prove valuable for the future development of the field of ECM research in general.

## 1. Introduction

Aging is a complex process that affects to some extent all tissues, organs, and body systems. Research into the molecular basis of aging has primarily focused on intracellular mechanisms, whereas many tissues in the body are largely composed of the extracellular matrix (ECM). The ECM is a highly dynamic three-dimensional structure that provides structural support for the cells and constantly undergoes enzymatic remodeling and non-enzymatic modification. Recent data demonstrate that quantitative and qualitative changes in the ECM affect diverse cell functions, including signaling, proliferation, migration, and differentiation. [1]. Due to their extremely long life, collagen and elastin, the major components of the ECM, are susceptible to pathological non-enzymatic modifications including glycation, carbonylation, and carbamylation [2]. Glycation products, also called advanced glycation end products (AGEs) play a significant role in the development of various diseases and are considered to be one of the key factors contributing to aging. The accumulation of AGEs has been found in various tissues and organs such as the skin, kidneys, bones, eyes, skeletal muscle, cartilage, arterial walls, and brain [3,4,5]. AGE accumulation leads to increased stiffness of the heart muscle that may contribute to the development of diastolic dysfunction [6,7]. There is evidence showing that the imbalance between matrix metalloproteinases (MMPs) and tissue inhibitors of matrix metalloproteinases (TIMPs) in the blood serum is associated with the development of myocardial fibrosis, leading to the development of cardiac dysfunction [8]. MMPs actively participate in ECM remodeling, degrading its components such as collagen, elastin, fibronectin, and proteoglycans [9]. Dysregulation of ECM remodeling contributes to altered intercellular communication, inflammaging, fibrosis, stem cell aging, and cellular senescence [2]. Increasing evidence indicates that the ECM plays an important role in tumor progression and metastasis [10,11]. Increased matrix stiffness has been shown to promote elements of epithelial–mesenchymal transition and also induce chemoresistance in pancreatic cancer cell lines [12]. Moreover, increased ECM rigidity is associated with the activation of Wnt/β-catenin signaling [13], the modulation of which is associated with many types of cancer [14]. In recent years, the role of the ECM in the development of sarcopenia, the age-related progressive loss of muscle mass and muscle function, has also been widely discussed [15]. A stiffened muscle ECM influences YAP/TAZ-mediated expression of matrix-associated proteins by fibroblasts, resulting in the decreased regenerative potential of muscle stem cells [16]. Age-related changes in the ECM composition play an important role in Alzheimer’s disease and other types of neurodegenerations [17,18,19]. In the brain, the ECM participates in various important brain functions such as diffusion regulation, synaptic plasticity, learning, and memory [20]. Thus, alterations in the structure and composition of the ECM, or the matrisome, are found in various types of tissues and accompany many diseases, including age-related diseases. However, in the literature, there is no structured and complete data set on age-related changes in ECM components, both at the protein level and at the level of gene expression.

The aim of this study was to identify matrisome genes differentially expressed with age in diverse human tissues. We used data from the Genotype-Tissue Expression (GTEx) project on seven tissues (blood, heart, brain, liver, kidneys, lungs, and muscle) grouped by age and sex. In addition, the list of matrisome genes from MatrisomeDB, the ECM-protein knowledge database containing in silico and in vivo data on human and mouse matrisomes, was used [21]. Considering that organs and tissues can age at different rates, it is hypothesized that tissue-specific changes in the ECM with age make a significant contribution to the aging pattern of these tissues and organs.

## 2. Materials and Methods

For the transcriptomic analysis, the GTEx tissue-specific RNA-Seq data was obtained from the GTEx Portal (count level, https://www.gtexportal.org/home/downloads/adult-gtex/bulk_tissue_expression (accessed on 23 March 2024)). Gene expression data from 2717 samples across 7 tissues (blood, brain, heart—left ventricle and atrial appendage, kidneys—cortex and medulla, liver, lungs, and skeletal muscle) were downloaded. The brain category included cortex, hippocampus, cerebellum, caudate, and putamen (basal ganglia) data. Data for each tissue were grouped by sex and age (20–39 years old group, 40–59 years old group, and 60–79 years old group). GTEx cohort characteristics by tissue are presented in Supplementary Table S1. GTEx annotation is available at https://storage.googleapis.com/adult-gtex/annotations/v8/metadata-files/GTEx_Analysis_v8_Annotations_SampleAttributesDS.txt (accessed on 23 March 2024).

Differential expression (DE) analysis was carried out in the R environment (v.3.6.3, Vienna, Austria) (R: The R Project for Statistical Computing, accessed on 3 March 2023; available online, https://www.r-project.org/) using the edgeR package (v.3.24.3, NSW, Australia). This software is designed for finding changes between two or more groups [22]. To normalize the obtained data, the TMM (Trimmed Mean of M-values, “calcNormFactors” edgeR function) method was applied with calculation of the CPM (counts per million, “cpm” edgeR function) considering the normalization coefficients [23]. The quasi-likelihood F-test (QLF) was used to evaluate the significance of differences in gene expression. The Benjamini–Hochberg adjustment for multiple testing was applied to *p*-values to calculate the false discovery rate (FDR). Moreover, in order to estimate the value of the change in gene expression during aging, the log2 fold-change (logFC) between the three age groups, as well as the logFC between young (20–39 years old) and old (60–79 years old) groups, were calculated. Obtained DE genes were filtered to only the core matrisome (*n* = 274) and matrisome-associated genes (*n* = 753), according to the list of human genes from MatrisomeDB (https://matrisomedb.org, accessed on 23 March 2024) [21]. In addition, DE matrisome genes were separated into categories: genes encoding collagens (*n* = 44), proteoglycans (*n* = 35), and ECM glycoproteins (*n* = 195) (core matrisome) and genes encoding ECM-affiliated proteins (*n* = 171), ECM regulators (*n* = 238), and secreted factors (*n* = 344) (matrisome-associated genes). Next, using the STRING database (Search Tool for the Retrieval of Interacting Genes/Proteins) (https://string-db.org, accessed on 23 March 2024) [24] we identified pathways which were enriched with DE core matrisome and matrisome-associated genes that passed an FDR threshold of 5% and with LogCPM > 0.5. Lists of upregulated and downregulated genes were used separately for each tissue.

## 3. Results

In the current study, we analyzed the age-related changes in matrisome gene expression profiles in various human tissues, in particular in the blood, brain, heart, kidneys, liver, lungs, and muscle. According to the results, most DE genes were found in the blood, lungs, and muscles (Table 1), the tissues with the largest number of cases represented in the study. Most of the DE matrisome genes showed an increase in expression with age in all studied tissues, with the exception of the liver. It is worth noting that DE genes that passed the FDR threshold were not detected in all tissues. In the heart, brain, and kidneys of females, there were no genes that passed an FDR threshold of 5%. As for the male group, genes with FDR < 0.05 were expressed in the brain only for *CLEC4G*, and in the kidneys only for *IL11*. Further in the text, when no FDR data are given, DE genes with *p*-value < 0.05 are presented.

Lists of DE genes between three age groups with information about genes belonging to particular categories are presented in Supplementary Table S2. By comparing gene expression profiles in tissues obtained from young (20–39 years old group) and old (60–79 years old group) individuals, we expectedly obtained more DE genes, the fold change of which was also higher (Supplementary Table S3) than in the comparisons of the three age groups.

Next, we identified the top upregulated and downregulated genes in each category of ECM and ECM-associated proteins: genes encoding ECM glycoproteins, collagens, and proteoglycans for the core matrisome (Table 2) and genes encoding ECM-affiliated proteins, ECM regulators, and secreted factors for matrisome-associated proteins (Supplementary Table S4).

**Figure 1:**
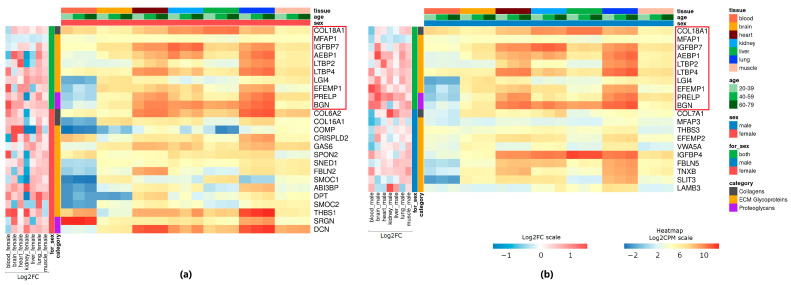
Commonly upregulated and downregulated matrisome genes with age in at least three tissues (LogCPM > 2, *p* < 0.05) in (**a**) female and (**b**) male groups. Common genes that changed expression with age in both sexes are circled in red.

To understand the similarities in matrisome gene expression patterns between tissue types, common genes were also selected that changed expression with age in at least three tissues (Figure 1, Supplementary Figure S1). Among core matrisome genes, *COL18A1*, *MFAP1*, *IGFBP7*, *AEBP1*, *LTBP2*, *LTBP4*, *LG14*, *EFEMP1*, *PRELP*, and *BGN* showed differential expression with age in both males and females (Figure 1). LTBPs (or latent transforming growth factor β binding proteins) are crucial to latent TGF-β location and activation. The latent complexes play an important role in the regulation of the TGF-β pathway, the alteration of which has been found in various age-related diseases [25]. Biglycan (BGN) and decorin (DCN) are major small leucine-rich proteoglycans expressed in connective tissues like the skin, bones, and tendons. Alterations in decorin and biglycan expression lead to structural abnormality in collagen fibrils and changes in the mechanical properties of the tissues [26,27]. The phenotype of the BGN/DCN double-knockout mice directly mimics the rare progeroid variant of human Ehlers–Danlos syndrome [26]. We found an increase in the expression of *BGN* with age in all tissues, except the kidneys in the male group. A statistically significant change in *DCN* expression (FDR < 0.05) was detected in the lungs of females, as well as in the muscle tissue of both sexes. As for the matrisome-associated genes, *FAM20B*, *CTSC*, *CTSS*, and *CLEC2B* were common DE genes across tissues in both sexes (Supplementary Figure S1). Other identified common DE genes had expression in a sex-specific manner, which was likely driven by the diverse biological characteristics in the context of sex [28]. For example, among common DE genes, we identified the sex-specific expression of *SERPING1*, which encodes C1-inhibitor (C1-INH). In the female group, the expression level of *SERPING1* was reduced in the liver and kidneys, while its expression was increased in the blood, brain, and muscle. Using sex-stratified gene regulatory network analysis, Hartman et al. identified SERPING1 as a potential key driver of coronary artery disease being more highly expressed in female smooth muscle cells [29].

**Figure 2:**
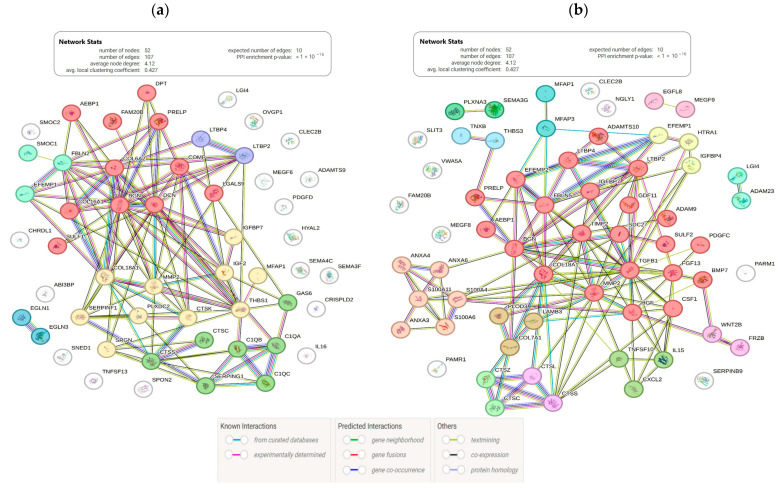
STRING networks (including physical interactions, co-expression, co-occurrence in databases, etc.) for genes whose expression was significantly associated with age in (**a**) female and (**b**) male groups. Every color corresponds to a cluster.

Additionally, using the STRING database, some information was obtained on the interactions of proteins encoded by these genes (Figure 2). Among the common DE genes (Figure 1, Figure 2), we found genes belonging to the cathepsins (*CTSC*, *CTSS*, *CTSK*, *CTSZ*, *CTSL*), MMPs (*MMP2*), TIMPs (*TIMP2*), and adamalysins (*ADAM9*, *ADAM23*, *ADAMTS10*) (Supplementary Figure S1, Figure 2) which a play significant role in ECM remodeling [30,31]. In the female group, genes (*C1QA*, *C1QB*, *C1QC*) related to the complement system were downregulated with age in the liver and kidneys and upregulated in the blood and brain. The complement system forms the core of the innate immune system and affects inflammation, metabolism, apoptosis, mitochondrial function, and the Wnt signaling pathway [32]. Among genes that demonstrated sex-specific alterations in expression during tissue aging, there were genes (*ANXA4*, *ANXA6*, *ANXA3*, *S100A11*, *S100A4*, *S100A6*) related to the “calcium-dependent protein binding” pathway.

In addition, using the STRING database, for each tissue we identified pathways which were enriched with DE genes (both core matrisome and matrisome-associated genes). For functional enrichment analysis, lists of upregulated and downregulated genes were used separately. “TGF-beta signaling pathway”, “focal adhesion”, “AGE-RAGE signaling pathway in diabetic complications”, “Hippo signaling pathway”, “PI3K-Akt signaling pathway”, and “pathways in cancer” were common statistically significant enriched pathways (FDR  <  0.05) with upregulated genes in all tissues studied. In muscle, among the pathways enriched with downregulated genes we identified, among others, “response to stress”, “response to hypoxia”, “regulation of proteolysis”, and “protein hydroxylation”. Downregulation of genes related to the “IL-17 signaling pathway” and “JAK-STAT signaling pathway” in the heart, downregulation of genes related to the “Toll-like receptor signaling pathway” in the liver, and alterations in expression genes of the “lamellar body” and “lung fibrosis” pathways in the lungs, were also observed.

## 4. Discussion

The aging ECM is characterized by impaired remodeling and affects the functioning of many tissues, contributing to the development of various pathological conditions [33]. The most well-known ECM-related tissues are collagen-rich tissues such as the skin, tendons, and bones. Among them, the most studied tissue from the point of view of aging is the skin. Many studies have been conducted confirming the pivotal role of ECM during both intrinsic and extrinsic skin aging (photoaging) [34,35]. In the skin, the ECM provides mechanical strength and acts as a barrier against the outside environment. In the bones and tendons ECM also provides mechanical properties, which allows the body to stand and to move [36,37]. However, as recent studies show, the functions of the ECM are much broader than structural and mechanical support to tissues. ECM is not limited to load-bearing organs but is present in all types of tissues and organs.

The current work focused on age-related matrisome transcriptome dynamics in normal human tissues such as the blood, heart, brain, liver, muscle, lungs, and kidneys. The largest number of genes differentially expressed with age was found in the muscle, lungs, blood, and heart. However, this is likely due more to the relatively small sample size of other studied tissues. On the other hand, it is worth taking into account the different rates of development of age-related changes in tissues, organs, and systems of organs, as well as differences in the composition and structure of the matrix of individual tissues and organs. For example, the ECM components of the brain are mainly synthesized by neurons and glial cells, and, unlike other tissues, the major components of the neural ECM are chondroitin sulfate proteoglycans (CSPGs), tenascin-R, and hyaluronic acid [38]. We detected a slight decrease in the expression of the *CSPG5* gene with age in the brain. Reduced expression of CSPGs has been reported to lead to the impairment of adult hippocampal neurogenesis [39] and may be involved in aging-related cognitive decline [40]. Expression of the *RELN* gene, encoding reelin, another ECM protein that is involved in maintaining hippocampal synaptic plasticity, was also reduced in aged brain tissues [41,42]. Several studies have shown that reelin deficiency may accelerate learning and memory impairment, which accompanies dementia and other aging-related diseases [43,44].

ECM aging is accompanied by an imbalance between the synthesis of ECM components and their proteolysis. As a result, the ECM gets stiffer, which intensifies age-related alterations, including the uncontrolled activation of fibrotic pathways in various tissues [45]. It is known that the prevalence of myocardial fibrosis and idiopathic pulmonary fibrosis dramatically increases with age [46]. Among age-related DE genes in the lungs, a number of genes involved in the “lung fibrosis” pathway were discovered. The results obtained for the lungs are partly consistent with the work of Ngassie et al., who have recently identified seven ECM proteins with higher expression in aged lung tissues at both gene and protein levels: COL1A1, COL6A1, COL6A2, COL14A1, FBLN2, LTBP4, and LUM [47]. We found a slight but statistically significant (FDR < 0.05) increase in the expression of *COL6A2*, *LTBP4*, and *LUM*, as well as genes encoding fibulins such as *FBLN1*, *FBLN7*, and *FBLN5*. Also, among the statistically significantly increased genes, genes (*LOX*, *LOXL4*, *LOXL1*, *LOXL2*) encoding lysyl oxidase (LOX) and LOX-like enzymes, which catalyze the cross-linking of elastin and collagen in the ECM and play a central role in ECM remodeling, were identified [48].

In the muscle, age-related differential expression of many genes (*SEMA3F*, *SEMA3G*, *SEMA6A*, *PLXNC1*, *SEMA4C*, *SEMA6D*, *PLXNB2*, *SEMA4A*, *SEMA6C*, *PLXNA3*, *SEMA5A*, *SEMA6B*, *SEMA3B* with FDR < 0.05) involved in the “semaphorin–plexin signaling pathway” was found. Recently, Fard et al. have shown that specific semaphorin molecules are involved in skeletal muscle regeneration and neuromuscular junction (NMJ) remodeling [49]. Defects in NMJ formation and defective muscle regeneration can lead to age-related skeletal muscle atrophy, also known as sarcopenia. In the muscle, as well as in the heart and lungs, an age-related increase in the expression of genes associated with the Hippo signaling pathway was detected. Indeed, it has been demonstrated that the age-related increase in matrix stiffness may impact YAP and TAZ activity, the prime mediators of the Hippo pathway, in fibroblasts and stem cells [16,50]. YAP/TAZ activity in fibroblasts contributes to the fibrogenic conversion of skeletal muscle, which in turn leads to further stiffening of the ECM [51]. Moreover, YAP/TAZ nuclear translocation and subsequent transcription of target genes participating in fibrosis development can be caused by the activation of Rho-ROCK through integrin-dependent signaling. According to the data of the present study, aging in the muscle, heart, and lungs leads to age-related differential expression of genes involved in the KEGG pathway “focal adhesion” and GO pathways such as “integrin binding” and “heparin binding”. In almost all studied tissues, the differential expression of genes in the TGF-β pathway, which is well known to be associated with the progression of fibrosis and is overactivated during aging, was found [52,53]. Accumulating evidence indicates that matrix stiffness can regulate fibrosis by controlling the integrin-dependent activation of TGF-β and the activation of non-canonical TGF-β signaling pathways [54].

Taken together, in this study we investigated age-associated matrisome gene expression profile changes occurring in normal human tissues. Different tissues have an ECM with a unique composition and topology. And while a comparison of tissues is useful, age-related differences specific to a tissue will also be important in understanding age-related diseases. The conducted analysis generated lists of DE matrisome genes with age in the blood, heart, brain, liver, kidneys, lungs, and muscle tissues sorted by categories: ECM glycoproteins, collagens, and proteoglycans for core matrisome genes and ECM-affiliated proteins, ECM regulators, and secreted factors for matrisome-associated genes. It was also revealed that there were sex-specific age-related alterations in the matrisome gene expression. At the same time, the sex imbalance in GTEx datasets and the lack of sufficient data for the brain, kidneys, and liver from younger individuals are limitations of this study. It is also worth remembering that ECM components undergo and assemble into complex supramolecular structures characterized by specific biophysical and biochemical properties. For example, it has been shown that cross-linking of collagen in the heart muscle can increase tissue stiffness without changes in total collagen content [55]. In this context, transcriptomic studies related to ECM should be approached with caution. On the other hand, one can assume that identifying the role of age-related changes in the gene expression of ECM genes is a necessary step both for understanding the basic biology of ECM aging in general and for solving applied problems of aging. Thus, the obtained results contribute to the understanding of tissue-specific changes in the ECM with age and can be useful to the scientific community.

## Figures and Tables

**Table 1:** Number of differentially expressed genes with age.

Tissue/Organ	Female				Male			
	Upregulated		Downregulated		Upregulated		Downregulated	
	C	MA	C	MA	C	MA	C	MA
Blood	29	99	17	79	33	129	27	98
Heart	27	21	0	15	43	87	10	50
Brain	19	35	6	25	7	19	13	25
Liver	8	23	7	36	4	15	16	55
Kidneys	0	15	1	8	11	26	10	18
Lung	78	99	7	49	106	194	10	107
Muscle	54	84	4	34	106	164	19	38

C—core matrisome, MA—matrisome-associated, *p*-value < 0.05, LogCPM > 0.

**Table 2:** The top 10 upregulated and downregulated DE core matrisome genes with age ranked by fold-change.

		Upregulated		Downregulated	
Tissue/Organ	Category	Female	Male	Female	Male
Blood	collagens	*COL6A2*	*COL6A2*	*COL9A3* *COL7A1*	*COL9A3* *COL7A1* *COL9A2* *COL18A1*
	ECM glycoproteins	*FGG* *FGB* *FGA* *SPP1* *SPON2* *THBS1* *LTBP4* *COLQ* *TNXB* *LAMB3*	*FGG* *VWCE* *THBS1* *FGA* *SPON2* *FGB* *COLQ* *LTBP4* *IGFBP4* *LTBP3*	*CRISPLD2* *TNFAIP6* *AEBP1* *MFAP3* *LRG1* *FGL2* *FBN2* *LAMC1* *THBS3* *MFAP1*	*TNFAIP6* *CRISPLD2* *PCOLCE2* *LRG1* *MFAP3* *VWA5A* *EFEMP2* *MFAP1* *THBS3* *GAS6*
	proteoglycans	*PRG2* *PRG3* *SPOCK2* *VCAN*	*PRG3* *PRG2* *SPOCK2* *VCAN*	*SRGN*	*SRGN*
Brain	collagens	*COL6A3* *COL1A1* *COL8A2* *COL1A2* *COL16A1*		*COL27A1*	*COL26A1*
	ECM glycoproteins	*VWA3A* *TINAGL1* *MGP* *EMILIN3* *LAMC3* *SMOC1* *LTBP2*	*EMILIN1* *LGI4* *VWCE*	*NPNT* *SMOC2*	*NELL1* *LGI2* *SLIT2* *MFAP3* *FBLN7* *NTNG1* *RELN*
	proteoglycans	*DCN*			
Heart	collagens	*COL14A1* *COL16A1*	*COL1A1* *COL3A1* *COL12A1* *COL5A1* *COL6A2* *COL16A1* *COL6A1*		
	ECM glycoproteins	*COMP* *LTBP2* *EFEMP1* *AEBP1* *SMOC2* *RELN* *IGFBP3* *FN1* *SRPX2* *SNED1*	*SPP1* *LTBP2* *IGFBP3* *TNC* *THBS4* *MXRA5* *SMOC2* *AEBP1* *EFEMP1* *SRPX2*		*ADIPOQ* *SMOC1* *THBS1* *VWA7* *THSD4* *MFAP1* *SRPX* *CRELD1*
	proteoglycans	*PRG4* *PRELP* *FMOD* *BGN*	*PRG4* *ASPN* *FMOD* *BGN* *OMD* *VCAN* *SRGN*		
Kidneys	collagens		*COL7A1*		*COL5A2*
	ECM glycoproteins		*FGG* *FGB* *FGA* *LAMB3* *LAMC2* *VTN* *NTN1*	*ADIPOQ*	*HMCN2* *NELL1* *LAMA1* *ECM1* *EFEMP2*
Liver	collagens	*COL7A1* *COL27A1*	*COL7A1* *COL5A3*	*COL4A1*	*COL4A1* *COL1A1* *COL4A2*
	ECM glycoproteins	*SMOC1* *SPON2* *IGFBP4*	*LAMB3*	*SPP1* *LAMC1* *VWA5A* *LAMB2*	*TGFBI* *NTN4* *CRELD1* *MFAP3* *LAMB2* *MMRN2* *MFAP1* *LAMA5*
	proteoglycans	*ASPN*	*VCAN*		*PRG* *HSPG2*
Lung	collagens	*COL11A2* *COL23A1* *COL9A2* *COL14A1* *COL6A6* *COL21A1* *COL4A6* *COL16A1* *COL18A1* *COL4A5*	*COL6A6* *COL13A1* *COL16A1* *COL21A1* *COL7A1* *COL18A1* *COL23A1* *COL12A1* *COL11A2* *COL4A1*	*COL24A1*	*COL6A5* *COL4A4*
	ECM glycoproteins	*DPT* *SMOC2* *IGFBP6* *TNFAIP6* *MFAP4* *AEBP1* *ELN* *MFAP5* *CRISPLD2* *MGP*	*COMP* *CTHRC1* *DPT* *TNR* *RSPO4* *ECM2* *SMOC2* *AEBP1* *MFAP4* *NTNG2*	*SPP1* *VWA1* *MFAP1*	*LGI3* *FGA* *DMBT1* *SPP1* *LRG1* *LAMB3* *LAMC2* *NTN4* *MFAP1* *VWA1*
	proteoglycans	*ACAN* *PRG4* *PRELP* *OMD* *ASPN* *DCN* *SRGN* *BGN* *PODN* *FMOD*	*ASPN* *ESM1* *ACAN* *HAPLN3* *PRG4* *PRELP* *BGN* *SRGN* *PODNL1* *OMD*		
Muscle	collagens	*COL21A1* *COL4A6* *COL4A3* *COL7A1* *COL8A1* *COL4A5* *COL6A3*	*COL1A1* *COL3A1* *COL1A2* *COL15A1* *COL5A1* *COL6A2* *COL8A1* *COL6A1* *COL7A1* *COL18A1*	*COL5A3*	
	ECM glycoproteins	*MFAP4* *PCOLCE* *DPT* *NPNT* *SPON2* *LGI4* *LTBP1* *FBLN2* *SNED1* *SLIT2*	*FNDC1* *THBS4* *NPNT* *PCOLCE* *MFAP4* *SVEP1* *DPT* *SPON2* *TNXB* *POSTN*	*MFAP3* *MFAP1*	*IGFBP5* *MFAP3* *SMOC1* *MFAP1*
	proteoglycans	*CHAD* *LUM* *FMOD* *HAPLN3* *PODN* *DCN* *SRGN*	*LUM* *CHAD* *ASPN* *BGN* *PODN* *VCAN* *HAPLN3* *FMOD* *DCN* *SRGN*		

*p*-value < 0.05, LogCPM > 2, genes which passed FDR < 0.05 are highlighted in red.

## Data Availability

All data generated or analyzed during this study are available within the article or upon request from the corresponding authors.

## Supplementary Materials

The following supporting information can be downloaded at: https://www.mdpi.com/article/10.3390/life14050593/s1, Figure S1: Commonly upregulated and downregulated matrisome-associated genes in (a) females (b) males; Table S1: GTEx cohort characteristics by tissue; Table S2: Age-related core matrisome transcriptome dynamics in the blood, brain, heart, kidneys, liver, lungs, and muscle; Table S3: Age-related matrisome-associated gene expression dynamics in the blood, brain, heart, kidneys, liver, lungs, and muscle; Table S4: The top 10 upregulated and downregulated matrisome-associated genes with age ranked by LogFC.
