# Supplementary material for: Matrisome Transcriptome Dynamics during Tissue Aging
Source: Life (Basel). 2024 May 7;14(5):593. doi: 10.3390/life14050593 (PMC11121957; doi:10.3390/life14050593)
Supplement: Supplementary file 1 [file life-14-00593-s001.zip › Supplementary Materials.pdf]

Communication

Matrisome Transcriptome Dynamics during Tissue Aging

Zulfiya G. Guvatova [1,2], Anastasiya A. Kobelyatskaya [1], Eveline R. Kudasheva [2], Elena A. Pudova [1], Elizaveta V. Bulavkina [1], Alexey V. Churov [2], Olga N. Tkacheva [2] and Alexey A. Moskalev [1,2,*]

[1] Engelhardt Institute of Molecular Biology, Russian Academy of Sciences, 119991 Moscow, Russia
[2] Russian Clinical Research Center for Gerontology, Pirogov Russian National Research Medical University, Ministry of Healthcare of the Russian Federation, 129226 Moscow, Russia
* Correspondence: amoskalev@list.ru

Abstract: The extracellular matrix (ECM) is a complex three-dimensional network of macromolecules that provides structural support for the cells and plays a significant role in tissue homeostasis and repair. Growing evidence indicates that dysregulation of ECM remodeling contributes to various pathological conditions in the body, including age-associated diseases. In this work, gene expression data of normal human tissues obtained from the Genotype-Tissue Expression project, as well as data from MatrisomeDB 2.0, the ECM-protein knowledge database, are used to estimate the age-dependent matrisome transcriptome dynamics in the blood, heart, brain, liver, kidneys, lungs, and muscle. Differential gene expression (DE) analysis revealed dozens of matrisome genes encoding both structural elements of the ECM and ECM-associated proteins, which had a tissue-specific expression profile with age. Among common DE genes that changed expression with age in at least three tissues, *COL18A1, MFAP1, IGFBP7, AEBP1, LTBP2, LTBP4, LG14, EFEMP1, PRELP, BGN, FAM20B, CTSC, CTSS*, and *CLEC2B* were observed. The findings of the study also reveal that there are sex-specific alterations during aging in the matrisome gene expression. Taken together, the results obtained in this work may help in understanding the role of the ECM in tissue aging and might prove valuable for the future development of the field of ECM research in general.

Keywords: aging; extracellular matrix; matrisome; RNA-Seq; tissue aging; transcriptome; gene expression

1. Supplementary Materials

Supplementary Table S1. GTEx cohort characteristics by tissue.

	Sex	Age group	Blood	Brain	Heart	Kidney	Liver	Lungs	Muscle
Cases, n	Male	20-39	92	2	17	7	18	50	93
		40-59	226	20	103	29	84	105	245
		60-79	183	27	83	30	59	140	205
	Female	20-39	44	3	11	2	6	23	39
		40-59	121	10	52	8	34	88	134
		60-79	89	8	34	9	25	72	87

Supplementary Table S4. The top 10 upregulated and downregulated matrisome-associated genes with age ranked by LogFC.

		Upregulated ↑		Downregulated ↓	
**Tissue/ Organ**	**Category**	**Female**	**Male**	**Female**	**Male**
**Blood**	ECM Regulators	*AMBP (1.79)*<br>*SERPINA3 (1.5)*<br>*HRG (1.34)*<br>*SERPINH1 (0.97)*<br>*CTSW (0.81)*<br>*SERPINB2 (0.77)*<br>*PLAU (0.62)*<br>*P4HA1 (0.57)*<br>*CTSG (0.57)*<br>*ADAMTS10 (0.51)*	*HRG (1.47)*<br>*SERPINH1 (0.91)*<br>*AMBP (0.89)*<br>*PLAU (0.82)*<br>*TGM3 (0.7)*<br>*CTSW (0.66)*<br>*CTSG (0.64)*<br>*SERPINB2 (0.63)*<br>*ELANE (0.53)*<br>*MMP19 (0.52)*	*PRSS2 (-0.9)*<br>*HPSE (-0.43)*<br>*MMP25 (-0.41)*<br>*EGLN1 (-0.38)*<br>*HYAL2 (-0.31)*<br>*ST14 (-0.27)*<br>*CTSK (-0.26)*<br>*ADAM17 (-0.26)*<br>*SERPINB8 (-0.25)*<br>*ADAM19 (-0.24)*	*HPSE (-0.55)*<br>*HYAL2 (-0.46)*<br>*SERPINB1 (-0.43)*<br>*ADAMTS2 (-0.42)*<br>*MMP25 (-0.41)*<br>*EGLN1 (-0.4)*<br>*CTSK (-0.37)*<br>*ADAM17 (-0.37)*<br>*CSTA (-0.37)*<br>*SERPINB8 (-0.36)*
	ECM-affiliated Proteins	*SDC2 (0.86)*<br>*C1QC (0.54)*<br>*C1QB (0.47)*<br>*C1QA (0.46)*<br>*CLC (0.44)*<br>*PLXNA3 (0.43)*<br>*CLEC2B (0.42)*<br>*CLEC11A (0.42)*<br>*SEMA4C (0.34)*<br>*ANXA6 (0.31)*	*CLC (0.71)*<br>*SDC2 (0.59)*<br>*PLXDC1 (0.54)*<br>*SDC4 (0.46)*<br>*PLXNA3 (0.45)*<br>*CLEC11A (0.42)*<br>*SEMA4C (0.4)*<br>*C1QC (0.36)*<br>*CLEC2B (0.36)*<br>*C1QA (0.35)*	*SFTPB (-1.02)*<br>*ANXA9 (-0.49)*<br>*CLEC4A (-0.36)*<br>*CLEC4E (-0.34)*<br>*SEMA4B (-0.34)*<br>*ANXA3 (-0.32)*<br>*PLXNA2 (-0.29)*<br>*CLEC12A (-0.29)*<br>*CLEC4D (-0.28)*<br>*PLXNC1 (-0.27)*	*CLEC4E (-0.67)*<br>*ANXA9 (-0.54)*<br>*CLEC4D (-0.53)*<br>*CLEC12A (-0.49)*<br>*ANXA3 (-0.48)*<br>*CLEC12B (-0.48)*<br>*ANXA1 (-0.41)*<br>*CLEC4A (-0.41)*<br>*CLEC1B (-0.39)*<br>*PLXNC1 (-0.38)*
	Secreted Factors	*IGF2 (1.29)*<br>*IFNG (1.24)*<br>*CCL3 (1.15)*<br>*CCL4 (1.03)*<br>*LEP (1.02)*<br>*LIF (0.98)*<br>*CCL4L2 (0.92)*<br>*CCL5 (0.81)*<br>*AREG (0.8)*<br>*VEGFA (0.8)*	*CCL3L3 (1.13)*<br>*IGF2 (1.12)*<br>*CCL3 (1.08)*<br>*CCL4L2 (1.05)*<br>*CCL4 (0.93)*<br>*TNFSF9 (0.93)*<br>*EREG (0.84)*<br>*XCL2 (0.81)*<br>*AREG (0.81)*<br>*PRL (0.76)*	*TNFSF10 (-0.53)*<br>*INHBB (-0.46)*<br>*IL1B (-0.44)*<br>*IL1RN (-0.43)*<br>*TNFSF14 (-0.42)*<br>*S100A4 (-0.34)*<br>*INSL3 (-0.34)*<br>*TGFA (-0.33)*<br>*MEGF9 (-0.32)*<br>*PPBP (-0.29)*	*TNFSF10 (-0.66)*<br>*PDGFC (-0.53)*<br>*S100A12 (-0.5)*<br>*S100A9 (-0.47)*<br>*S100A8 (-0.47)*<br>*TNFSF14 (-0.46)*<br>*TGFA (-0.45)*<br>*S100A4 (-0.44)*<br>*MEGF9 (-0.41)*<br>*IL1RN (-0.4)*
**Brain**	ECM Regulators	*F13A1 (0.7)*<br>*ADAM28 (0.63)*<br>*CTSS (0.61)*<br>*CPAMD8 (0.38)*<br>*MMP2 (0.29)*<br>*ADAM33 (0.25)*<br>*ADAMTSL2 (0.19)*	*MMP14 (0.27)*<br>*EGLN3 (0.21)*<br>*CSTB (0.16)*<br>*CTSD (0.12)*	*MASP1 (-0.32)*<br>*ADAM12 (-0.27)*<br>*ADAMTS8 (-0.25)*<br>*EGLN3 (-0.2)*<br>*ADAM17 (-0.16)*<br>*EGLN1 (-0.11)*	*SERPINI1 (-0.28)*<br>*ADAM22 (-0.17)*<br>*FAM20B (-0.16)*<br>*ADAM9 (-0.15)*
	ECM-affiliated Proteins	*CLEC7A (1.06)*<br>*C1QB (0.87)*<br>*C1QA (0.83)*<br>*C1QC (0.82)*<br>*LMAN1L (0.78)*<br>*SFTPC (0.32)*<br>*SEMA4C (0.13)*	*C1QTNF1 (0.32)*<br>*LGALS1 (0.19)*<br>*SDC3 (0.12)*	*C1QTNF3 (-0.36)*<br>*ELFN1 (-0.36)*<br>*CSPG5 (-0.28)*<br>*SDC2 (-0.25)*<br>*PLXNC1 (-0.17)*<br>*SEMA6D (-0.16)*	*CLEC4G (-0.81)*<br>*C1QL3 (-0.43)*<br>*PLXNC1 (-0.19)*<br>*ELFN2 (-0.17)*
	Secreted Factors	*CRLF1 (0.64)*<br>*FGF17 (0.3)*	*CXCL10 (2.61)*<br>*GH1 (2.29)*<br>*PRL (1.55)*<br>*CSF1 (0.3)*<br>*S100A6 (0.23)*<br>*S100B (0.23)*<br>*PDGFA (0.16)*	*CBLN4 (-0.58)*<br>*CXCL14 (-0.54)*<br>*WIF1 (-0.54)*<br>*CHRDL1 (-0.5)*<br>*WNT7A (-0.44)*<br>*WNT7B (-0.36)*<br>*PTN (-0.29)*<br>*BRINP2 (-0.26)*<br>*BRINP3 (-0.26)*<br>*MEGF10 (-0.17)*	*CRHBP (-0.95)*<br>*CBLN4 (-0.49)*<br>*FGF12 (-0.34)*<br>*FGF13 (-0.34)*<br>*KITLG (-0.22)*<br>*FGF14 (-0.16)*<br>*NRG3 (-0.13)*<br>*MEGF9 (-0.1)*
**Heart**	ECM Regulators	*LOXL1 (0.31)*<br>*HTRA1 (0.18)*<br>*MMP14 (0.16)*	*PRSS2 (0.82)*<br>*SLPI (0.63)*<br>*LOX (0.46)*<br>*SERPINH1 (0.34)*<br>*PAMR1 (0.32)*	*C17orf58 (-0.23)*	*ADAM23 (-0.35)*<br>*ADAMTS15 (-0.28)*<br>*ADAM11 (-0.27)*<br>*SERPINB1 (-0.24)*<br>*TGM2 (-0.23)*

			LOXL1 (0.27)<br>LOXL2 (0.26)<br>ITIH5 (0.25)<br>HTRA1 (0.25)<br>ADAMTS5 (0.22)		HYAL1 (-0.15)<br>NGLY1 (-0.14)<br>ADAM9 (-0.14)<br>CTSL (-0.14)<br>P4HA2 (-0.13)
	ECM-affiliated Proteins	COLEC12 (0.26)<br>PLXDC2 (0.2)	ITLN1 (0.86)<br>CLEC2B (0.39)<br>CLEC1A (0.29)<br>PLXDC2 (0.27)<br>GPC6 (0.26)<br>LGALS9 (0.26)<br>CLEC2D (0.21)<br>PLXDC1 (0.21)<br>ANXA3 (0.2)<br>SEMA3G (0.19)	SEMA6D (-0.2)	GPC4 (-0.33)<br>PLXNA4 (-0.18)<br>PARM1 (-0.17)<br>SEMA6D (-0.17)<br>GPC1 (-0.14)<br>PLXNB3 (-0.13)<br>LGALS8 (-0.11)<br>ANXA6 (-0.11)<br>SEMA4D (-0.11)<br>ANXA11 (-0.11)
	Secreted Factors	SFRP4 (0.66)<br>WNT9A (0.52)<br>MEGF6 (0.5)<br>FRZB (0.43)<br>CHRDL1 (0.43)<br>IGF2 (0.26)<br>WNT2B (0.17)	FRZB (0.52)<br>CCL21 (0.47)<br>VEGFC (0.4)<br>SFRP4 (0.4)<br>CXCL3 (0.36)<br>ANGPTL4 (0.36)<br>BMP6 (0.29)<br>BMP2 (0.28)<br>PTN (0.27)<br>CX3CL1 (0.25)	CSF3 (-2.57)<br>CXCL8 (-1.73)<br>CXCL1 (-1.18)<br>IL6 (-1)<br>INHBA (-0.43)<br>CRHBP (-0.4)<br>EGF (-0.24)<br>NRTN (-0.13)	INHBA (-0.59)<br>LIF (-0.54)<br>CXCL8 (-0.53)<br>EGF (-0.41)<br>FST (-0.4)<br>FGF7 (-0.34)<br>INHA (-0.28)<br>SCUBE3 (-0.24)<br>BMP7 (-0.24)<br>BMP5 (-0.22)
**Kidney**	ECM Regulators	ADAMTS4 (1.75)<br>EGLN3 (0.76)<br>ADAMTS9 (0.6)	SERPINE1 (0.7)<br>PAPPA (0.33)	TIMP4	SERPINE2 (-0.71)<br>ADAM33 (-0.56)<br>ADAMTS15 (-0.45)<br>CTSF (-0.18)
	ECM-affiliated Proteins	CLEC2B (0.92)<br>LGALS3 (0.57)<br>MUC1 (0.47)<br>MUC20 (0.38)	REG1A (1.25)<br>MUC6 (0.67)<br>CLEC2B (0.35)<br>OVGP1 (0.25)	LGALS9 (-0.34)<br>SEMA6A (-0.32)<br>C1QTNF6 (-0.23)	MUC15 (-0.49)<br>GPC3 (-0.44)<br>ELFN1 (-0.3)<br>PLXNB3 (-0.27)
	Secreted Factors	FGF7 (1.35)<br>FGF11 (0.95)<br>S100A14 (0.76)	IL11 (1.72)<br>IL6 (1.23)<br>ANGPTL4 (1.03)<br>CXCL8 (0.87)<br>CXCL2 (0.81)<br>INHBB (0.77)<br>CXCL10 (0.72)<br>S100A2 (0.62)<br>BMP2 (0.46)<br>CLCF1 (0.28)		BMP6 (-0.47)<br>FRZB (-0.4)<br>BMP4 (-0.3)<br>CTF1 (-0.26)<br>TNFSF12 (-0.16)
**Liver**	ECM Regulators	ADAM19 (0.38)<br>ADAMTSL4 (0.22)	EGLN3 (0.66)<br>C17orf58 (0.13)	SERPINE2 (-0.53)<br>PLG (-0.41)<br>CTSS (-0.39)<br>CTSC (-0.3)<br>HABP2 (-0.24)<br>SERPINA10 (-0.23)<br>CTSZ (-0.17)	LOXL4 (-0.54)<br>LOXL2 (-0.36)<br>CTSS (-0.32)<br>SERPINB1 (-0.31)<br>CTSL (-0.29)<br>SERPINA1 (-0.29)<br>ADAM9 (-0.25)<br>HYAL2 (-0.24)<br>AGT (-0.22)<br>P4HA2 (-0.21)
	ECM-affiliated Proteins	FREM2 (0.92)<br>COLEC11 (0.33)<br>GPC1 (0.2)<br>SEMA4C (0.18)	FREM2 (0.68)<br>LGALS4 (0.68)	C1QC (-0.94)<br>MUC5B (-0.94)<br>C1QB (-0.86)<br>FCN1 (-0.82)<br>C1QA (-0.77)<br>SDC3 (-0.48)<br>LGALS9 (-0.44)<br>SEMA3F (-0.3)<br>PLXNA1 (-0.3)<br>ANXA4 (-0.19)	C1QC (-0.58)<br>LGALS9 (-0.41)<br>SEMA6B (-0.35)<br>SEMA3F (-0.33)<br>ANXA1 (-0.32)<br>SDC3 (-0.28)<br>PLXNA1 (-0.2)<br>PLXND1 (-0.2)
	Secreted Factors	HGFAC (0.38)<br>VEGFB (0.37)<br>ANGPTL6 (0.37)<br>GDF7 (0.34)	CXCL13 (2.1)<br>GDF15 (0.9)<br>CXCL2 (0.36)<br>S100A14 (0.34)	CXCL8 (-0.87)<br>S100A4 (-0.42)<br>S100A11 (-0.33)<br>TGFB1 (-0.33)	LIF (-0.58)<br>PDGFA (-0.57)<br>IL1RN (-0.52)<br>INHBA (-0.51)

		NRTN (0.3)<br>CHRD (0.28)<br>TGFA (0.23)<br>TNFSF13 (0.21)<br>PDGFC (0.19)<br>TNFSF12 (0.19)	S100A16 (0.17)<br>HCFC2 (0.08)	CSF1 (-0.24)	TNFSF14 (-0.49)<br>TGFB3 (-0.48)<br>CCL2 (-0.48)<br>FSTL3 (-0.41)<br>SCUBE1 (-0.38)<br>S100A11 (-0.33)
**Lungs**	ECM Regulators	PI3 (0.97)<br>MMP10 (0.73)<br>ADAMTS9 (0.5)<br>SERPINE1 (0.47)<br>LOX (0.46)<br>ADAMTSL1 (0.42)<br>EGLN3 (0.41)<br>ITIH3 (0.4)<br>SERPINA3 (0.4)<br>HTRA3 (0.33)	MASP1 (0.55)<br>ADAMTS4 (0.48)<br>PAMR1 (0.47)<br>SERPINB9 (0.41)<br>MMP1 (0.41)<br>LOX (0.4)<br>ADAM12 (0.39)<br>EGLN3 (0.39)<br>SERPINE1 (0.37)<br>ADAMTS9 (0.34)	ELANE (-0.48)<br>SERPINA1 (-0.38)<br>CTSH (-0.37)<br>MMP15 (-0.37)<br>ST14 (-0.29)<br>CSTB (-0.26)<br>CTSD (-0.25)<br>HPSE (-0.24)<br>CTSS (-0.22)<br>MMP28 (-0.22)	PI3 (-0.69)<br>CTSH (-0.64)<br>SLPI (-0.64)<br>MMP7 (-0.47)<br>SERPINA1 (-0.47)<br>SERPINF2 (-0.44)<br>MMP24 (-0.42)<br>CTSE (-0.41)<br>ST14 (-0.37)<br>MMP28 (-0.31)
	ECM-affiliated Proteins	SEMA3D (0.34)<br>OVGP1 (0.32)<br>MUC3A (0.31)<br>C1QTNF1 (0.31)<br>CSPG4 (0.28)<br>GPC3 (0.27)<br>CLEC2D (0.26)<br>CLEC4E (0.25)<br>GPC6 (0.23)<br>C1QTNF3 (0.21)	GREM1 (1.06)<br>ITLN1 (0.54)<br>CLEC1A (0.49)<br>OVGP1 (0.4)<br>SEMA3A (0.34)<br>CLEC2B (0.34)<br>SEMA3D (0.33)<br>CLEC2D (0.31)<br>SEMA7A (0.28)<br>GPC3 (0.26)	CSPG5 (-0.45)<br>SFTPD (-0.39)<br>CLEC10A (-0.39)<br>CLEC5A (-0.38)<br>SFTPB (-0.37)<br>FREM2 (-0.37)<br>MUC1 (-0.35)<br>SFTA2 (-0.3)<br>LGALS9 (-0.23)<br>CLEC12A (-0.22)	SFTPA1 (-0.86)<br>SFTPC (-0.83)<br>SFTPA2 (-0.83)<br>SFTPD (-0.81)<br>FREM2 (-0.75)<br>MUC1 (-0.66)<br>SFTPB (-0.64)<br>SFTA3 (-0.51)<br>SFTA2 (-0.5)<br>CSPG5 (-0.42)
	Secreted Factors	FRZB (0.73)<br>SFRP1 (0.66)<br>IGF2 (0.59)<br>FST (0.57)<br>TGFB3 (0.51)<br>FGF14 (0.46)<br>CXCL14 (0.44)<br>FGF2 (0.43)<br>PDGFD (0.42)<br>S100A3 (0.4)	CXCL9 (0.62)<br>FGF14 (0.62)<br>FRZB (0.61)<br>CXCL14 (0.57)<br>CXCL10 (0.55)<br>FGF1 (0.53)<br>PDGFD (0.52)<br>PTN (0.46)<br>FGF7 (0.44)<br>S100A3 (0.42)	HHIP (-0.58)<br>CCL13 (-0.44)<br>TNFSF9 (-0.4)<br>S100A14 (-0.37)<br>WNT7A (-0.22)<br>TNFSF13 (-0.2)<br>S100A11 (-0.14)	HHIP (-0.92)<br>WIF1 (-0.68)<br>S100A14 (-0.61)<br>WNT7B (-0.58)<br>MST1L (-0.47)<br>CCL13 (-0.42)<br>CXCL2 (-0.39)<br>SHH (-0.36)<br>CXCL1 (-0.29)<br>WNT7A (-0.27)
**Muscle**	ECM Regulators	ADAMTS17 (0.4)<br>ADAM23 (0.39)<br>ADAMTS9 (0.32)<br>PLAT (0.28)<br>ADAMTSL4 (0.25)<br>HTRA1 (0.25)<br>MMP2 (0.24)<br>CTSK (0.23)<br>LOX (0.23)<br>SULF2 (0.19)	ADAMTSL2 (0.63)<br>LOXL4 (0.43)<br>HTRA1 (0.41)<br>MMP2 (0.4)<br>CTSK (0.39)<br>LOXL2 (0.34)<br>PLAT (0.33)<br>ADAMTS2 (0.3)<br>ADAMTSL4 (0.29)<br>ADAMTS9 (0.29)	SERPINA5 (-0.69)<br>MASP1 (-0.41)<br>KAZALD1 (-0.32)<br>P4HA1 (-0.27)<br>CTSD (-0.23)<br>CST3 (-0.23)<br>FAM20B (-0.22)<br>SERPINB6 (-0.22)<br>NGLY1 (-0.18)<br>P4HA2 (-0.16)	SERPINA5 (-0.63)<br>MMP3 (-0.43)<br>SERPINB1 (-0.38)<br>PCSK6 (-0.28)<br>FAM20B (-0.24)<br>NGLY1 (-0.23)<br>EGLN1 (-0.2)<br>SERPINB6 (-0.2)<br>CPAMD8 (-0.19)<br>ADAM17 (-0.17)
	ECM-affiliated Proteins	FREM2 (0.34)<br>SEMA6D (0.28)<br>SEMA3F (0.25)<br>PLXNB2 (0.24)<br>MUC20 (0.23)<br>SEMA4A (0.21)<br>SEMA6B (0.2)<br>SDC2 (0.18)<br>SEMA3G (0.17)<br>PLXNA3 (0.17)	PLXNB2 (0.37)<br>SEMA3F (0.32)<br>FREM2 (0.32)<br>CLEC3B (0.3)<br>SEMA3G (0.28)<br>ANXA1 (0.26)<br>LGALS3 (0.23)<br>SEMA6D (0.23)<br>PLXNA3 (0.23)<br>MUC20 (0.22)	LMAN1 (-0.11)	GPC1 (-0.14)<br>ANXA2 (-0.14)<br>LMAN1 (-0.13)<br>ANXA7 (-0.06)
	Secreted Factors	FST (0.73)<br>AREG (0.63)<br>SFRP2 (0.53)<br>IGF2 (0.48)<br>ANGPT2 (0.42)<br>GDNF (0.37)<br>PDGFC (0.33)<br>CHRDL1 (0.33)<br>IL15 (0.31)<br>CXCL14 (0.3)	SFRP2 (0.94)<br>FST (0.61)<br>SFRP4 (0.6)<br>IGF2 (0.55)<br>AREG (0.47)<br>SFRP1 (0.42)<br>IL17D (0.34)<br>FGF7 (0.33)<br>CHRDL1 (0.33)<br>WNT11 (0.32)	IL34 (-0.42)<br>ANGPTL4 (-0.39)<br>S100A9 (-0.32)<br>S100A8 (-0.25)<br>ZFP91 (-0.15)<br>VEGFB (-0.15)<br>HCFC1 (-0.14)<br>ANGPTL1 (-0.12)<br>MEGF9 (-0.09)	IL34 (-0.65)<br>ANGPTL7 (-0.42)<br>NRG2 (-0.33)<br>S100A9 (-0.32)<br>TGFB3 (-0.21)<br>S100A8 (-0.18)<br>ZFP91 (-0.18)<br>HCFC1 (-0.12)<br>IL16 (-0.1)<br>MEGF9 (-0.09)

* *p*-value < 0.05, LogCPM > 2, genes passed FDR < 0.05 are highlighted in red

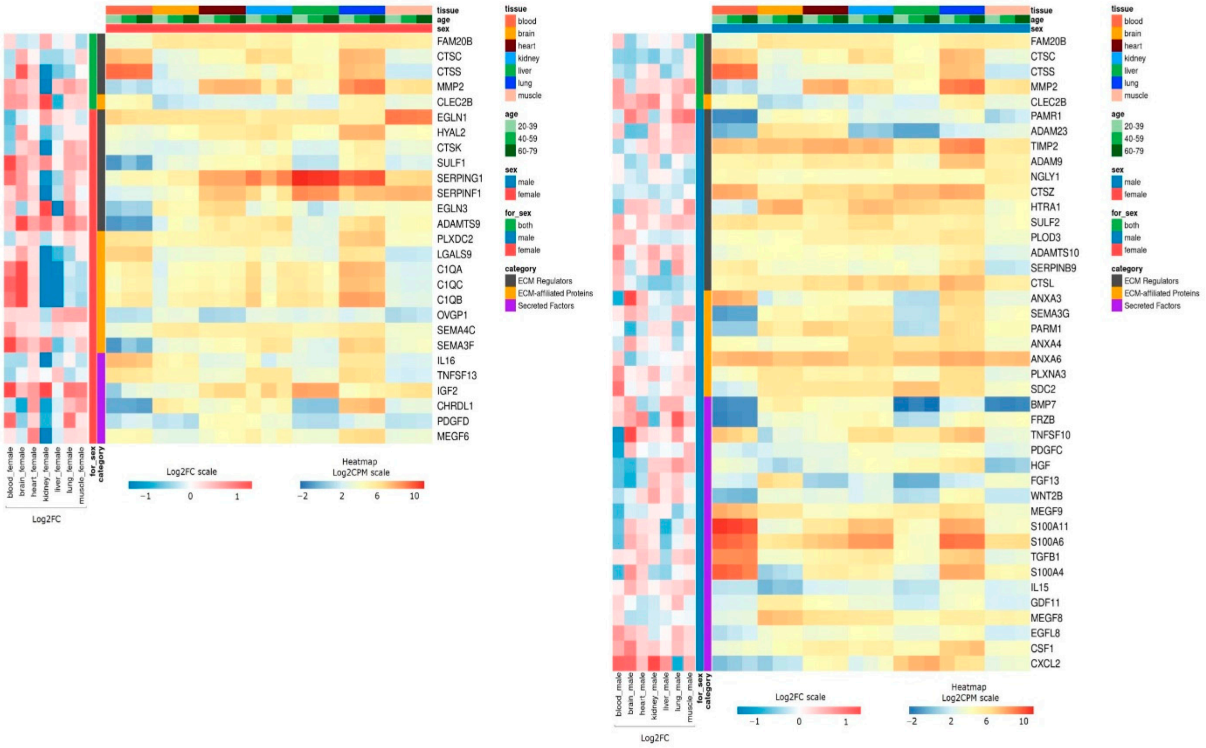

(**a**) (**b**)

Supplementary Figure S1. Commonly upregulated and downregulated matrisome-associated genes with age in at least three tissues (LogCPM > 2, $p < 0.05$) in (**a**) females (**b**) males.
